# Synergistic combination of orally available safe-in-man pleconaril, AG7404, and mindeudesivir inhibits enterovirus infections in human cell and organoid cultures

**DOI:** 10.1007/s00018-025-05581-4

**Published:** 2025-01-23

**Authors:** Erlend Ravlo, Aleksandr Ianevski, Jørn-Ove Schjølberg, Vanessa Solvang, Rabina Dumaru, Hilde Lysvand, Jacqueline Hankinson, Markus Vähä-Koskela, Sanna Vainionpää, Anni Varhe, Hanna Seppänen, Teemu Smura, Xin Wang, Yining Wang, Pengfei Li, Qiuwei Pan, Knut Dahl-Jorgensen, Lars Krogvold, Oleksandr Kamyshnyi, Hans-Johnny Schjelderup Nilsen, Inger Johanne Haugen, Erling Høyer, Jan Egil Afset, Valentyn Oksenych, Angel S. Galabov, Adelina Stoyanova, Vivian Lam, Barbara van Loon, Valeria Lulla, Magnar Bjørås, Denis E. Kainov

**Affiliations:** 1 Department of Clinical and Molecular Medicine (IKOM), Norwegian University of Science and Technology, Trondheim, 7028 Norway; 2 Department of Microbiology, Oslo University Hospital and University of Oslo, Oslo, 0372 Norway; 3 Department of Pathology, University of Cambridge, Cambridge, CB21QP UK; 4 Institute for Molecular Medicine FIMM, Helsinki Institute for Life Science, University of Helsinki, Helsinki, 00014 Finland; 5 Translational Cancer Medicine Research Program, Faculty of Medicine, University of Helsinki, Helsinki, 00014 Finland; 6 iCAN Digital Precision Cancer Medicine Flagship, Helsinki, 00014 Finland; 7 Department of Surgery, Helsinki University Hospital, Helsinki, Finland; 8 Department of Virology, University of Helsinki, Helsinki, 00014 Finland; 9 Clinical Microbiology, Helsinki University Hospital, HUS Diagnostic Center, University of Helsinki, Helsinki, 00029 Finland; 10 Department of Gastroenterology and Hepatology, Erasmus MC-University Medical Center, Rotterdam, 3015 Netherlands; 11 Faculty of Medicine, University of Oslo, Oslo, 0318 Norway; 12 Division of Paediatric and Adolescent Medicine, Oslo University Hospital, Oslo, 0424 Norway; 13 Department of Microbiology, Virology, and Immunology, I. Horbachevsky Ternopil National Medical University, Ternopil, 46001 Ukraine; 14 Department of Medical Microbiology, Clinic for Laboratory Medicine, St. Olavs Hospital, Trondheim, 7028 Norway; 15 Broegelmann Research Laboratory, Department of Clinical Science, University of Bergen, Bergen, 5021 Norway; 16 The Stephan Angeloff Institute of Microbiology, Bulgarian Academy of Sciences, Sofia, 1113 Bulgaria; 17 LHL, Jessheim, 2067 Norway; 18 Centre for Embryology and Healthy Development (CRESCO), University of Oslo, Oslo, 0373 Norway

**Keywords:** Enterovirus, Broad-spectrum antivirals, Antiviral drug combination, Drug synergy

## Abstract

Enteroviruses can infect various human organs, causing diseases such as meningitis, the common cold, hand-foot-and-mouth disease, myocarditis, pancreatitis, hepatitis, poliomyelitis, sepsis, and type 1 diabetes. Currently, there are no approved treatments for enterovirus infections. In this study, we identified a synergistic combination of orally available, safe-in-man pleconaril, AG7404, and mindeudesivir, that at non-toxic concentrations effectively inhibited enterovirus replication in human cell and organoid cultures. Importantly, the cocktail did not alter glucose and insulin levels in the culture medium of pancreatic β-cells and preserved the contraction rhythm of infected heart organoids. These findings highlight a promising drug cocktail for further preclinical studies and clinical trials targeting a broad range of enterovirus-mediated diseases.

**Supplementary Information:**

The online version contains supplementary material available at 10.1007/s00018-025-05581-4.

## Introduction

**Fig. 1:**
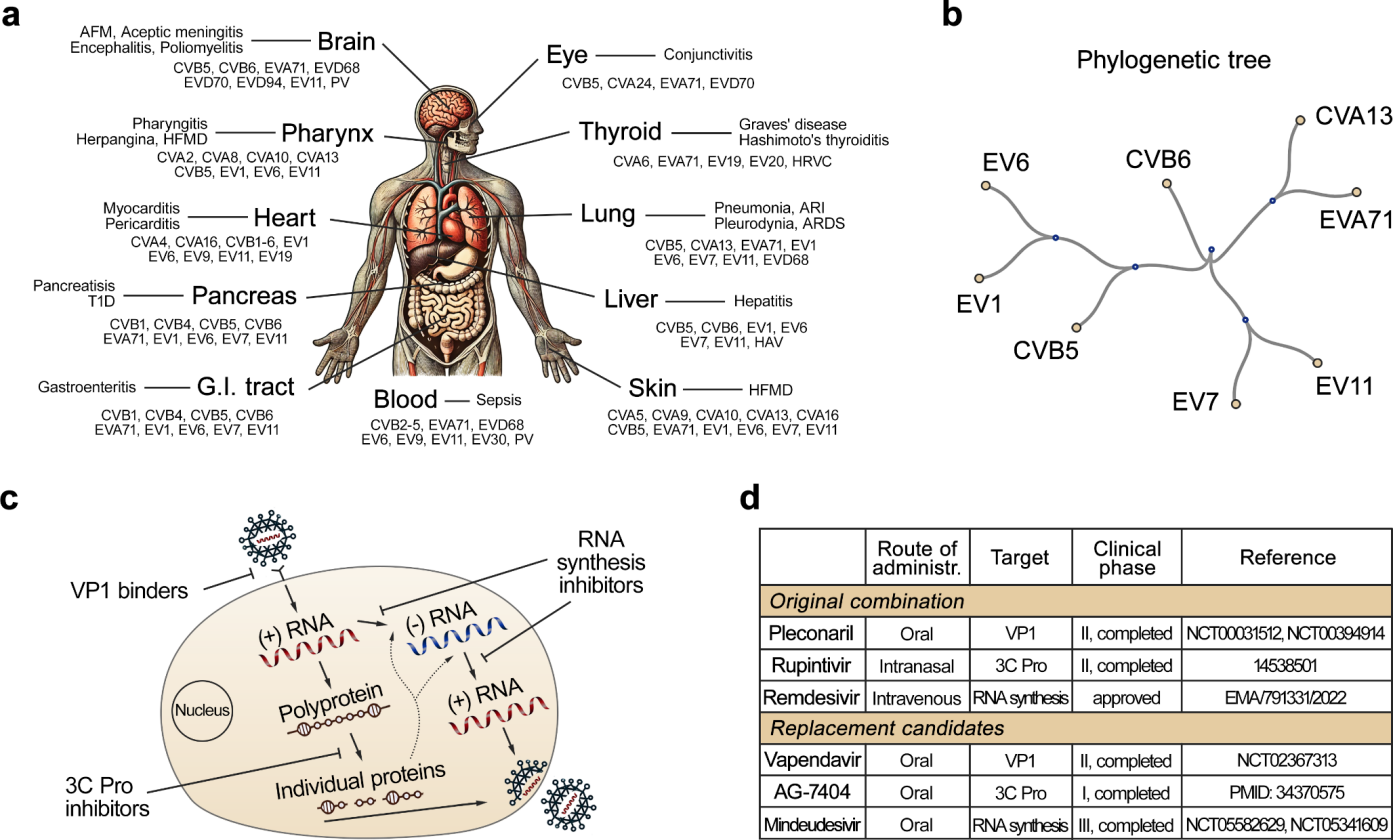
Orally available triple drug combination for potential treatment of picornavirus infections. **(a)** Examples of picornaviruses, target organs and associated diseases. **(b)** Phylogenetic tree of enteroviruses used in our study. **(c)** The life cycle of picornavirus and inhibitors. **(d)** Example of published synergistic anti-enterovirus combination and orally available compound substitutes

Picornaviruses are globally prevalent pathogens responsible for a wide range of diseases (Fig. 1a). These diseases can range from the common cold to severe conditions such as meningitis, myocarditis, pancreatitis, hand-foot-and-mouth disease (HFMD), sepsis, and poliomyelitis [1]. Additionally, the association of picornaviruses with chronic diseases such as asthma, allergies, and type 1 diabetes (T1D) underscores their significant clinical impact. Notably, infections with certain coxsackieviruses (CV) and echoviruses (EV) have been implicated in triggering T1D in children [2, 3].

Enteroviruses are a subgroup of picornaviruses. The Enterovirus genus within the *Picornaviridae* family includes 14 species. Recent outbreaks of echovirus 11 (EV11) from *E. betacoxsackie* have caused sepsis and death in neonates in Europe [4]. In 2023, Enterovirus A71 (EVA71) from *E. alphacoxsackie* caused a severe HFMD outbreak in Asia [5]. Additionally, CVA13 from *E. coxsackiepol* caused HFMD outbreak in Africa [6]. Notably, these and other enteroviruses share high sequence similarity and life cycle (Fig. 1b, c). Despite the widespread impact of enteroviral infections, there is a lack of approved anti-enteroviral drugs [7, 8]. Recent efforts have led to some progress in the development of virus inhibitors [9, 10]. However, the advancement of these inhibitors faced substantial hurdles, including concerns over a low coverage of enteroviruses, the rapid emergence of drug-resistant virus strains and drug toxicity, particularly in children.

To tackle some of these issues, antiviral drugs are combined into cocktails [11, 12]. Such cocktails were developed for treatment of HIV and HCV infections [7, 11]. Recently we began the development of such a cocktail against enterovirus infections. We showed that a combination of pleconaril, rupintrivir, and remdesivir inhibit replication of EV1, EV6, EV11, CVB5 in human lung A549 cell culture and EVA71 in intestinal organoids [13]. Pleconaril targets viral VP1 protein and prevents the virus entry into host cells, rupintrivir inhibits viral 3 C protease responsible for the processing of viral polyprotein, whereas remdesivir attenuates replication and transcription of viral RNA (Fig. 1c). Importantly, the combination delayed the emergence of antiviral drug resistance, distinguishing it from monotherapy or two-drug cocktails. Notably, the low doses of antiviral agents in the synergistic combination were less cytotoxic.

However, only pleconaril can be administered orally, while rupintrivir is delivered as an intranasal spray and remdesivir via intravenous infusion. This complicates the administration of the combination of pleconaril, rupintrivir and remdesivir to patients. The possibility of orally administering analogues of rupintrivir and remdesivir, such as AG7404 (AG-7404, V-7404) and mindeudesivir (VV116), respectively, highlights the potential for further pre-clinical development of such cocktail (Fig. 1d) [14–17]. Here we optimized the cocktail by combining orally available, safe-in-man pleconaril, AG7404, and mindeudesivir. The combination inhibited the replication of CVA13, CVB5, CVB6, EV1, EV6, EV7, and EV11 in human lung A549 and HE, eye RPE, cervical HeLa, skeletal RD, pancreatic MiaPaca-2 (MP-2) and β-cells, without detectable cytotoxicity. The combination also protected human pancreatic, eye, lung, heart and brain organoids from EV1-, EV11-, or CVB5-mediated cell death. Thus, we discovered combination of orally available small-molecules suitable for further animal studies and clinical trials against a broad range of enterovirus infections.

## Materials and methods

### Small molecules, viruses and cells

Supplementary Table S1 lists compounds used in the study, their suppliers, and catalogue numbers. To obtain 10 mM stock solutions, compounds were dissolved in dimethyl sulfoxide (DMSO; Sigma-Aldrich, Germany) or water. The solutions were stored at -20 °C until use. Supplementary Table S2 lists viruses used in the study, and their sources. EV1, EV6, EV11, CVB5 were amplified in a monolayer of A549 cells, EVA71 was amplified in Vero cells, CVB6 and CVA13 were amplified in HeLa cells, and EV7 in RD cells. Virus stocks were stored at − 80 °C. Supplementary TableS lists cells used in the study, and their sources. A549, MIA PaCa-2 (MP-2), HE, RD, HeLa and Vero cells were grown in Dulbecco’s Modified Eagle’s medium (DMEM; Gibco, Paisley, Scotland) supplemented with 100 U/mL penicillin and 100 µg/ml streptomycin mixture (pen/strep; Lonza, Cologne, Germany), 4.5 g/L (= 25 mmol/L) glucose, 1 mM L-glutamine, and 10% heat-inactivated fetal bovine serum (FBS; Lonza, Cologne, Germany). RPE cells were grown in DMEM-F12 supplemented with 10% FBS, pen/strep. All cells were cultured at 37ºC with 5% CO2, 95% humidity and passaged using 0.05% (v/v) Trypsin/EDTA (Gibco). Cells were tested mycoplasma negative throughout the work (MycoAlert Mycoplasma Detection Kit, Lonza). EndoC-BH5 cells (Human cell design) were maintained in Ultib1 EndoC-BH serum free medium (Human cell design) at 37 °C with 5% CO2, and 95% humidity.

### Virus infection and median tissue culture infectious dose calculation (TCID50)

The infection of A549, MP-2, HE and RPE cells was done in growth media containing 0.2% bovine serum albumin (BSA; Sigma) at a multiplicity of infection (moi) 0.1. The infection of RD, HeLa and Vero cells was done in growth media containing 1% FBS at an moi 0.1. The infection of EndoC-BH5 cells was done in DMEM containing 4.5 g/L glucose, 1 mM L-glutamine, 0.2% BSA and pen/strep at an moi 0.1. Viral sample (1 µL) at seven 10x dilutions was added to 99 µL of virus growth medium. The mixture was added to 96-well plates containing approximately 4 × 10^4^ A549 cells/well. At 48 h post infection, cell viability was measured with Cell Titer Glo (CTG; Promega) assay and a median tissue culture infectious dose 50 (TCID_50_) was calculated as described previously [18].

### RT-qPCRs

RNA was isolated using the RNAse kit (Qiagen) or NAxtra kit [19]. The isolated RNA was then reverse transcribed into cDNA using the SuperScript™ II Reverse Transcriptase synthesis kit (Thermo Fisher). Quantitative PCR (qPCR) was performed on the CFX Connect Real-Time PCR Detection System (Bio-Rad) using SYBR Green and virus-specific primers EV_fw: TAGTCCTCCGGCCCCTGAATGC and EV_rv: CCAATCCATAGCTATATGG. Viral RNA copy numbers were calculated using a standard curve analysis.

Alternatively, total RNA was isolated using Macherey–Nagel NucleoSpin^®^ RNA II kit (Bioke, Leiden, The Netherlands). cDNA was synthesized by using a cDNA synthesis kit (TaKaRa Bio, Inc., Shiga, Japan). Real-time PCR reactions were performed with PowerTrack™ SYBR Green Master Mix for qPCR (Applied Biosystems, Austin, USA) on a QuantStudio™ 3 Real-Time PCR System (Thermo Fisher Scientific LifeSciences). Glyceraldehyde 3-phosphate dehydrogenase (GAPDH) gene was used as housekeeping gene. Relative gene expression was analyzed using 2^−∆∆CT^ method [20]. Target gene expression was normalized to GAPDH using the formula:

1 $$\:\varDelta\:\varDelta\:\text{C}\text{T}\hspace{0.17em}=\hspace{0.17em}\varDelta\:\text{C}\text{T}\text{s}\text{a}\text{m}\text{p}\text{l}\text{e}\hspace{0.17em}-\hspace{0.17em}\varDelta\:\text{C}\text{T}\text{c}\text{o}\text{n}\text{t}\text{r}\text{o}\text{l}$$

where ∆CT = CT [target gene] − CT[GAPDH]. Template control and reverse transcriptase control were included in all RT-qPCR experiments. The primers used are GAPDH_fw: GTCTCCTCTGACTTCAACAGCG, GAPDH_rv: ACCACCCTGTTGCTGTAGCCAA, EV1_fw: TCCTCCGGCCCCGTGA, and EV1_rv: RATTGTCACCATAAGCAGCCA.

### Cell viability and death assays

The cell viability and death were measured using CellTiter-Glo (CTG), Alamar blue (Invitrogen, DAL1100, 1:10 dilution in AEM), and CellToxGreen assays (CTxG; Promega #G9241, G8741).

### Measurments of glucose and human insulin levels

Accu-Chek Mobile Blood Glucometer with test cassettes was used to measure glucose levels in cell culture medium (Roche). An enzyme-linked immunosorbent assay (ELISA) kit (Invitrogen) was used for the quantitative detection of human insulin levels in cell culture medium.

### Live cell imaging

Virus-induced cytotoxicity of RD, HeLa and Vero cells was assessed by live-cell imaging using an Incucyte SX5, an automated phase-contrast and fluorescence microscope within a humidified incubator, using Cytotox NIR Dye (Sartorius) at 1:1,000 dilution added directly during virus infection. At 3-hour intervals, 3 images per well were taken and used to estimate NIR-positive cells per cell using the integrated software. For each experiment, the data were plotted as NIR object count per image.

### Calculation of drug sensitivity scores

Drugs were added to cells in 5-fold serial dilutions. Cells were infected with viruses (moi 0.1) or mock. The cell viability was measured using CTG assay after 48 h of infection. A drug sensitivity score (DSS) was calculated as a normalized version of the standard area under dose–response curve (AUC), with the baseline noise subtracted, and the normalized maximal response at the highest concentration (often corresponding to off-target toxicity):

2 $$\:DSS=\frac{AUC-t({x}_{max}-{x}_{min})}{\left(100-t\right)({x}_{max}-{x}_{min}){\text{log}}_{10}Amin}$$,

where activity threshold *t* equals 10%, and DSS is in the 0–50 range [13, 21–23]. The difference (DDSS) between DSS (virus) and DSS (mock) was also calculated.

### Drug synergy calculations

Drugs were added to cells in 5-fold serial dilutions. Cells were infected with viruses (moi 0.1) or mock. The cell viability was measured using CTG after 48 h of infection. To test whether the drug combinations act synergistically, the observed responses were compared with expected combination responses. The expected responses were calculated based on the Bliss reference model using SynergyFinder version 3 to reveal whether changes in drug concentrations are associated with substantial increases in potency and efficacy [24]. Synergy scores were quantified as average excess response due to drug interactions for the most synergistic 4 × 4 × 4 dose-windows in dose–response matrices (i.e., 10% of cell survival beyond the expected additivity between single drugs represents a synergy score of 10).

### Human organoid cultures

*Pancreatic organoids.* Pancreatic cancer organoid cultures from two patients PO34T and PO80T have been described [25]. Organoids were maintained in matrigel domes and complete feeding medium [26]. Organoids were digested by TrypLE Express and Dispase to single cells and 2000 cells were seeded in 10 µl 4 mg/ml matrigel per well in 384-well plates (PhenoPlate 384, Revvity #6057302). After 20 min at 37 °C, 15 µl feeding medium was added on top of the matrigel and cells were cultured for 3–4 days to obtain pancreatic organoids.

*Airway organoids.* To produce human airway organoids (hAOs), adult lung tissues were obtained from tumor-free material of lung of cancer patient. hAOs were cultured in airway organoid expansion medium (AEM), based on advanced DMEM/F12 (Invitrogen), supplemented with 1% penicillin/streptomycin (Life Technologies), 1 M HEPES (Life Technologies), 200 mM Ultra glutamine (Life Technologies), 2% (vol/vol) of B27 (Gibco), 1.25 mM N-acetylcysteine (Sigma-Aldrich), 10 mM Nicotinamide (Sigma-Aldrich), 10% (vol/vol) of R-spondin-1 (conditioned medium), 10% (vol/vol) of Noggin (conditioned medium), 100 ng/ml FGF10 (Peprotech), 25 ng/ml FGF7 (Peprotech), 1 µM SB202190 (Tocris), 500 nM A83-01 (Tocris) and 10 µM Y27632 (Sigma-Aldrich).

*Brain organoids.* The brain organoids were generated from human induced pluripotent stem cells (iPSCs) according to published protocol with few modifications [27]. The dense, intact, and single spheroids of ≥ 250 µM were transferred to ultra-low attachment 96 well plate (CLS7007) on Day 7. On Day 9 of neural differentiation spheroids were transferred to 24 well plate (Corning, 3472). For the first week of transfer, half of OLIG3 media was changed every 3rd day. Later, two thirds of the media was changed every third day until day 30. By Day 30, the organoids were expressing neural progenitor markers.

*Retinal and heart organoids.* To generate retinal and heart organoids, human dermal fibroblasts were first reprogrammed into iPSCs using CytoTune™-iPS 2.0 Sendai Reprogramming Kit (ThermoFisher Scientific, A16517) following the manufacturer’s instructions. The iPSCs were maintained in Essential 8™ medium (ThermoFisher Scientific, A1517001) and routinely passaged every 4 to 5 days with 0.5 mM EDTA (ThermoFisher Scientific, 15575020). Retinal and heart organoids were generated from the iPSCs using modified versions of the published protocols [28, 29], respectively. The modifications include alterations to medium composition, differentiation mediators and timing of their addition, and initial seeding density during aggregation. Additionally, orbital shaking was employed throughout the differentiation and maintenance to increase nutrient uptake. On day 20 and day 53, post-differentiation induction, the retinal and cardiac organoids, respectively, were collected for the viral infection assay.

### Drug efficacy test in organoids

*Pancreatic organoids.* Compounds (0.1 µM pleconaril, 5 µM AG7404, and 5 µM mindeudesivir) and their combinations were introduced by acoustic dispenser (LabCyte Echo 525) and 1000 PFU of virus was added in 10 µL of feeding medium per well for a final volume of 35 µL per well. Estimated multiplicity of infection based on organoid doubling rate: 1 to 5. After 72 h, 10 µL of supernatant per well was carefully withdrawn for virus titration. CTG reagent (25 µL) was added per well. After 10 min at RT luminescence was read on the Agilent Cytation 5 plate reader/imager.

*Airway organoids.* Organoids were harvested from matrigel, treated with 0.1 µM pleconaril, 5 µM AG7404, 5 µM mindeudesivir and their combinations and infected with EV1 virus particles (moi = 2000 TCID_50_/organoid) for 1 h. Organoids were then washed by DMEM/F12 to thoroughly remove unabsorbed viruses. Organoids were embedded in matrigel and maintained in AEM with drugs at 37 °C with 5% CO_2_. Organoids and culture supernatant were collected for further analysis.

*Retinal*,* heart*,* and brain organoids.* One organoid was placed in each well in an ultralow attachment 96-well plate, with a total of 100 µl of media per well. Compounds (0.1 µM pleconaril, 1 µM AG7404, and 10 µM mindeudesivir) and their combinations were introduced and virus (20000 PFU/mL) or mock was added. After 2.5 h the media was replaced with fresh media containing compounds. After 72 h, 30 µl of media of each well was taken for virus detection by RT-qPCR. The contractions of heart organoids were monitored using microscope. The retinal organoids were sonicated, and ATP content (cell viability) was measured by CTG assay.

### Live organoid imaging

Pancreatic organoids were imaged with 2,5× objective on the Agilent Cytation 5 plate reader/imager. Retinal organoids were imaged using Olympus CKX53 microscope with 10x objective and an Olympus DP28 camera. Brain organoids were imaged using Evos cell imaging system with 4× objective and fluorescent filters (ThermoFisher Scientific). Heart organoids were imaged and filmed using Olympus CKX53 microscope with 10x brightfield objective and an Olympus UC90 camera. The resulting movies were then processed using CONTRACTIONWAVE software, which enabled the quantification of contractile dynamics across different organoid regions [30]. This method allows for the extraction of detailed contraction patterns and amplitude information, providing critical insights into the functional characteristics of the heart organoids under various experimental conditions.

## Results

### Synergistic combination of orally available, safe-in-man pleconaril, AG7404, and mindeudesivir inhibits replication of a several enteroviruses in human cell cultures

To identify orally available, safe-in-human analogues of rupintrivir (a 3 C protease inhibitor) and remdesivir (an RNA synthesis inhibitor), we reviewed our integrative drugvirus.info data portal along with recent studies from PubMed, ClinicalTrials, and DrugBank websites [31]. We identified several potential analogues, including AG7404 (a 3 C protease inhibitor), and mindeudesivir, obeldesivir, LY2334737, favipiravir, ribavirin, sofosbuvir, and molnupiravir (all RNA synthesis inhibitors). Initially, we tested these small molecules, as well as the orally available vapendavir (a VP1 binder), against CVB5 in human lung A549 cells. We determined the difference between DSS of virus and mock (ΔDSS). Vapendavir, AG7404, and mindeudesivir partially rescued CVB5-infected cells from death, but their ΔDSS were lower compared to pleconaril, rupintrivir, and remdesivir (Fig. S1, S2).

**Fig. 2:**
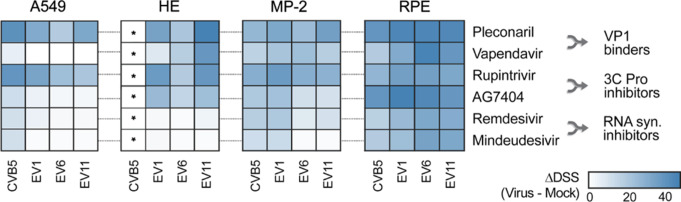
Comparison of antiviral activities of selected VP1 binders, 3 C protease and RNA synthesis inhibitors in cell cultures. Human A549, HE, MiaPaca-2 and RPE cells were treated for with increasing concentrations of compounds and infected with the CVB5, EV1, EV6, EV11 (moi 0.1) or mock. After 48 h, cell viability was determined using a CTG assay. Drug sensitivity scores (DSS) and their differences (DDSS) were calculated and shown as heatmaps (* - not determined because HE cells were not susceptible for CVB5 infection)

Next, we compared the ΔDSS of vapendavir and pleconaril, AG7404 and rupintrivir, and mindeudesivir and remdesivir pairs in human retinal RPE, pancreatic MP-2, and lung HE and A549 cell lines which were differentially susceptible to CVB5, EV1, EV6, and EV11 infections (Fig. S3). Vapendavir was found to be less effective than pleconaril, while AG7404 and mindeudesivir demonstrated moderate efficacy compared to rupintrivir and remdesivir, respectively (Fig. S4-S7, Fig. 2a). Based on these results, we selected a combination of the orally available pleconaril, AG7404, and mindeudesivir for further experiments.

We performed a time-of-addition experiment in A549 cells using CVB5 (Fig. S8). The results showed that the combination of pleconaril, AG7404, and mindeudesivir can be added between 2 h before and 12 h after infection and still effectively inhibit viral replication. This is due to the drugs’ ability to target different stages of viral replication..

**Fig. 3:**
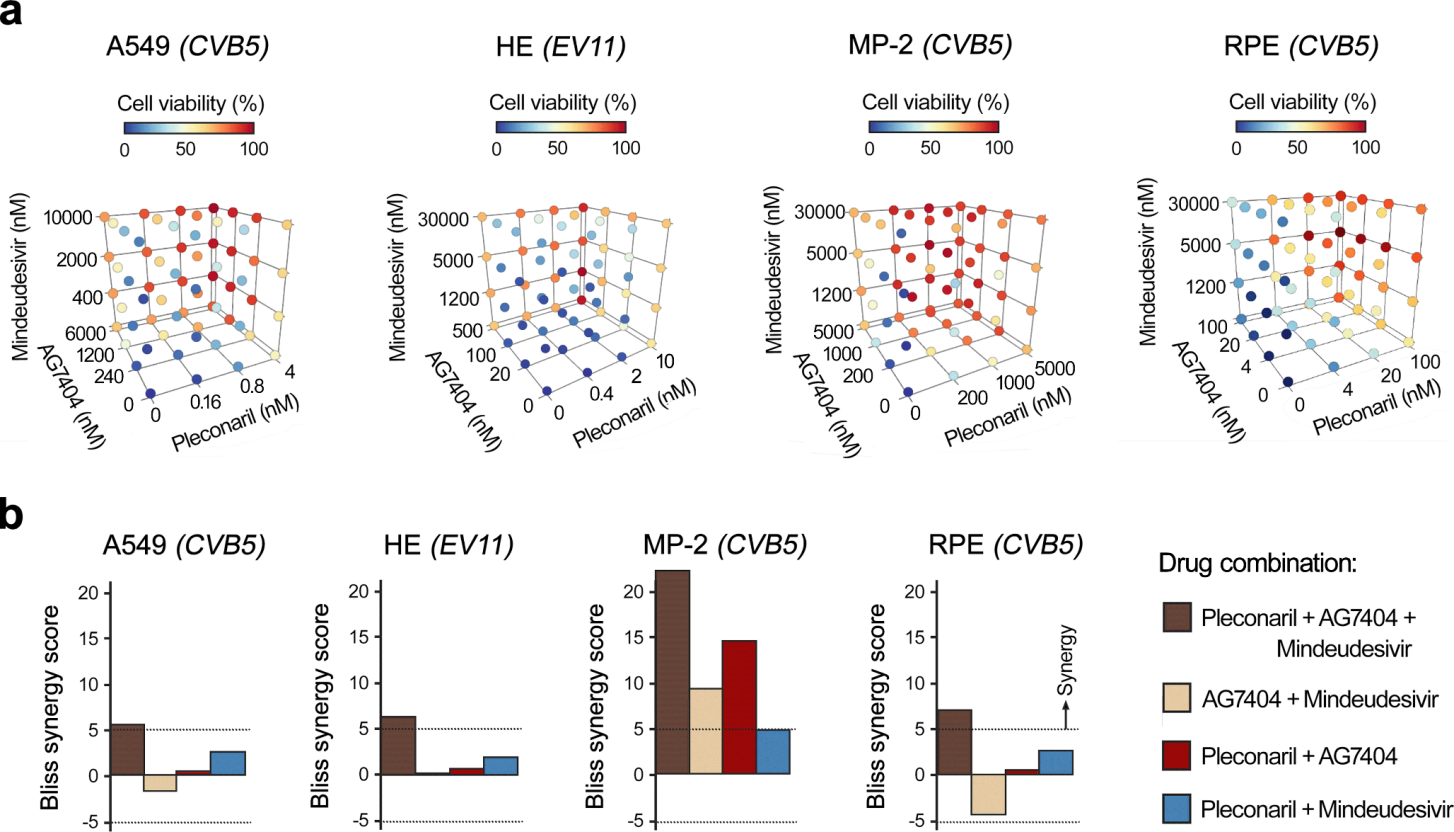
Anti-enteroviral effect of combination of pleconaril, AG7404 and mindeudesivir in cell cultures. **(a)** Cells were treated with increasing concentrations of pleconaril, AG7404, mindeudesivir or their combinations, and infected with enterovirus (moi 0.1). After 48 h, cell viability was determined using a CTG assay. The 6 × 6 × 6 interaction landscapes were obtained for the combination. **(b)** Bliss synergy scores were calculated for most synergistic areas (MSA) and plotted

We tested the combination of pleconaril, AG7404, and mindeudesivir against CVB5 in A549, HE, MP-2, and RPE cells. We obtained interaction landscapes of the compounds and calculated Bliss synergy scores (Fig. S9-S12, Fig. 3a, b). The Bliss scores indicated that the triple combination was synergistic/additive (i.e., reduced concentrations of the small molecules were needed to achieve the antiviral effect compared to double-drug combinations).

The combination was also tested against CVB6 and CVA13 in human cervical HeLa cells, EV7 in human skeletal RD cells, and EVA71 in monkey kidney Vero cells. The cocktail protected human HeLa and RD cells against CVA13, CVB6, and EV7 for 72 h, but not monkey Vero cells against EVA71 (Fig. S13). Notably, in contrast to the triple drug combination, pleconaril and the pleconaril-mindeudesivir combination were not able to protect HeLa cells after 45 h of CVB6 infection. This could be associated with CVB6-acquired pleconaril resistance [13].

**Fig. 4:**
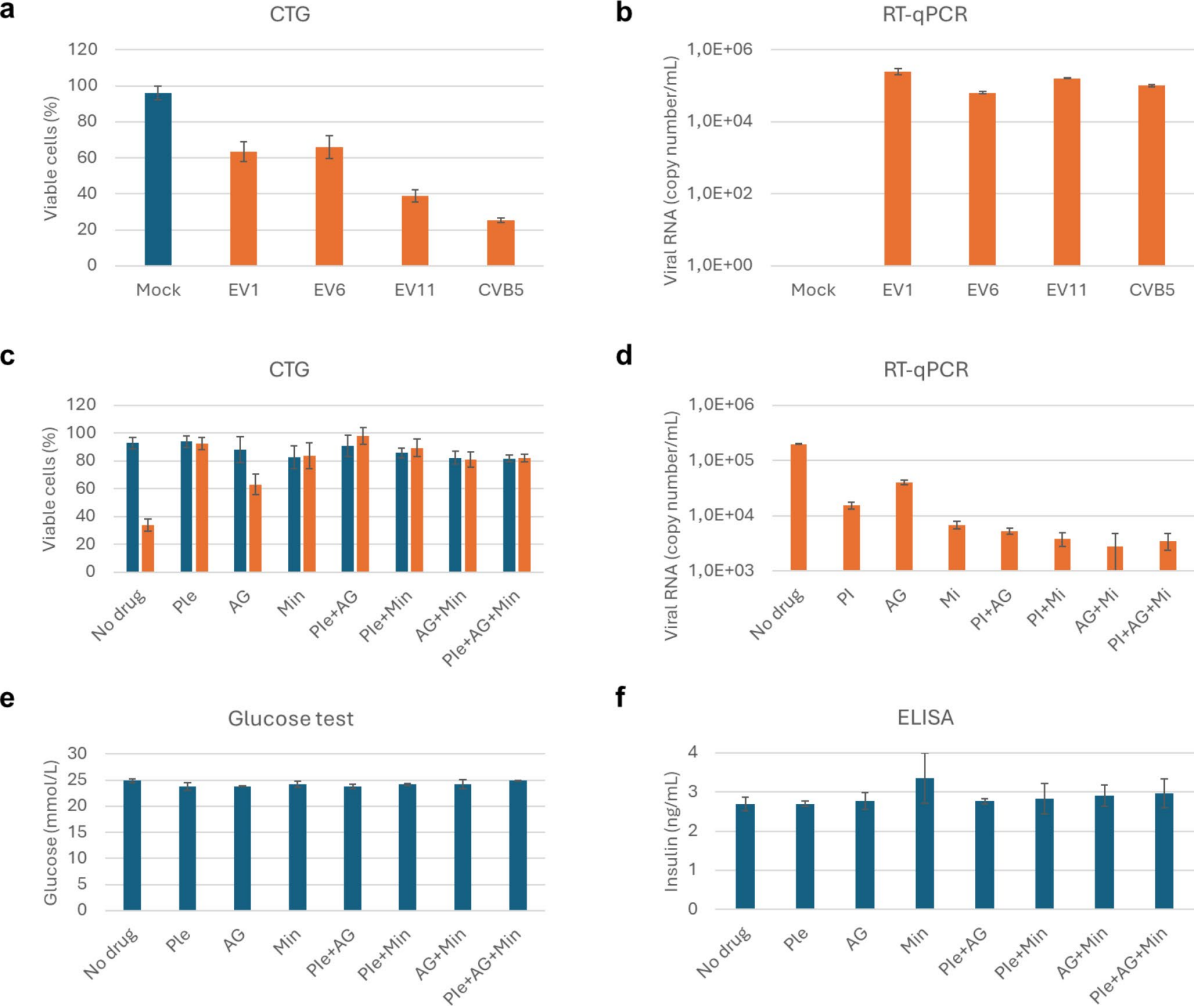
Antiviral efficacy of combination of pleconaril, AG7404 and mindeudesivir in EndoC-βH5 cells. (**a**) Viability of mock- and virus- (moi 0.1) infected cells at 48 hpi was determined by CTG assay. (**b**) RT-qPCR analysis of viral RNA isolated from media of virus-infected cells. (**c**) Cells were treated with 0.1 µM pleconaril, 1 µM AG7404, 10 µM mindeudesivir or their combinations for and infected with the CVB5 (orange) or mock (blue). After 1 h of infection, the media was replaced with fresh media containing the drugs but not the virus. After 48 h, viability of the cells was determined using a CTG assay. (**d**) Cells were treated as in (c) and CVB5-infected. RT-qPCR analysis of viral RNA was performed. (**e**) Cells were treated as in (c). Glucose levels were measured in the cell media using glucometer. (**f**) Cells were treated as in (c). Human iso-insulin was measured using ELISA. (a-f) Mean ± SD, *n* = 3

It has been demonstrated that enteroviruses destroy insulin-producing β- and other cells in the pancreas and trigger T1D [3, 32, 33]. Treatment with a combination of pleconaril and ribavirin has been shown to preserve residual insulin production in children and adolescents with new-onset T1D [33]. However, ribavirin was ineffective against CVB5, and CVB6 rapidly became resistant to pleconaril in vitro. We hypothesized that combination of pleconaril, AG7404, and mindeudesivir could protect b-cells from virus-mediated cells more efficiently than pleconaril and ribavirin. We evaluated the combination of pleconaril, AG7404, and mindeudesivir in human EndoC-βH5 cells, which closely mimic native pancreatic β-cells with physiological insulin secretion [34], and were susceptible to EV1, EV6, EV11 and CVB5 infection (Fig. 4a, b). We treated the cells with fixed doses of pleconaril, AG7404, mindeudesivir, and their combinations, followed by infection with CVB5. This treatment rescued the cells from virus-mediated death and significantly attenuated virus replication (Fig. 4c, d). Additionally, the drug treatment did not affect cell viability, glucose, or insulin levels (Fig. 4c, e, f).

Thus, we identified a synergistic combination of orally available, safe-in-human pleconaril, AG7404, and mindeudesivir that inhibits a broad range of enterovirus infections in human cell cultures.

### Combination of pleconaril, AG7404, and mindeudesivir inhibits replication of enteroviruses in human organoids

**Fig. 5:**
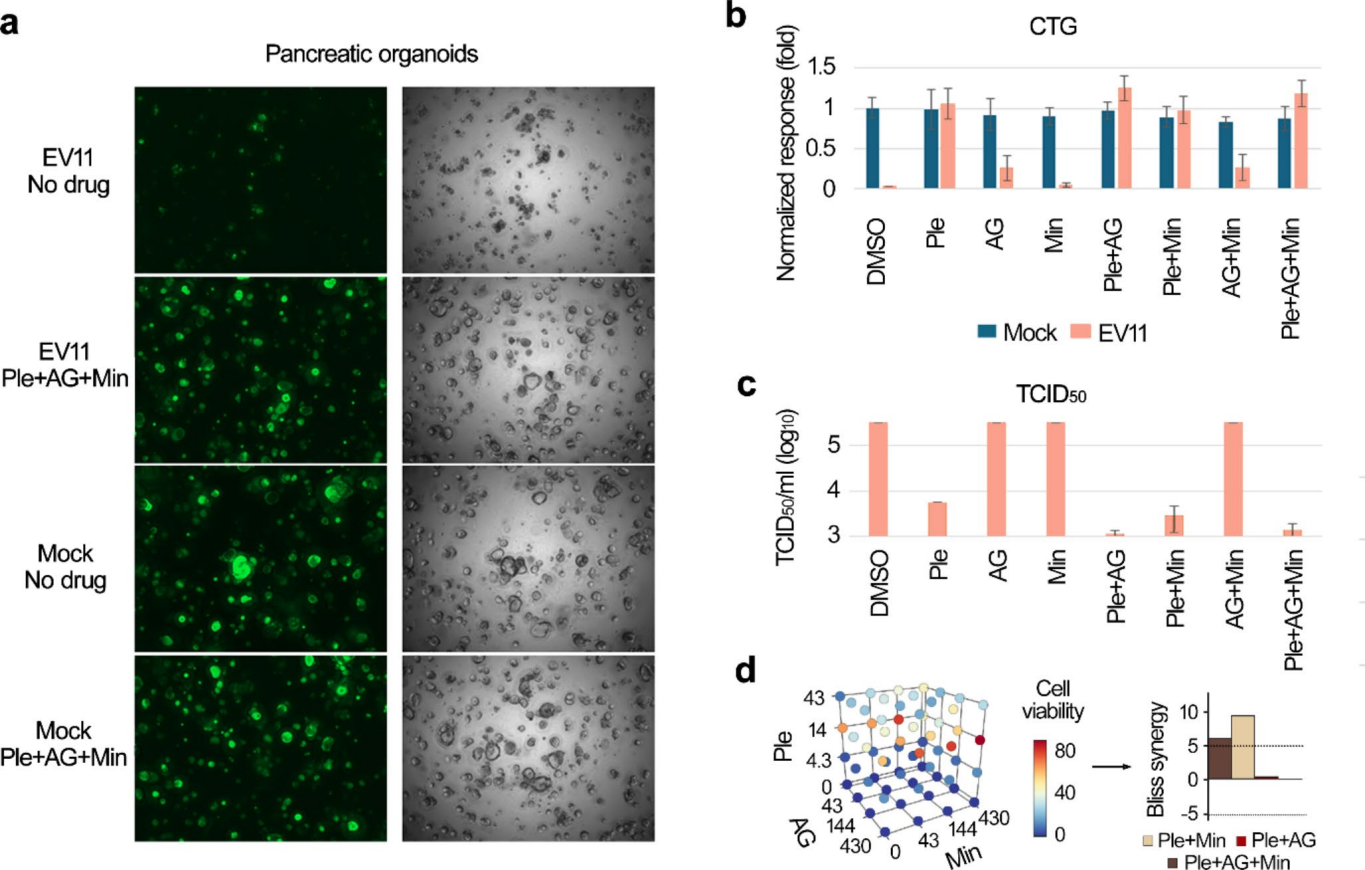
Effect of pleconaril, AG7404, and mindeudesivir combination on EV11- and mock-infected eGFP-expressing pancreatic organoids. (**a**) Organoids were differentiated for 4 days, treated with combination of 0.1 µM pleconaril, 5 µM AG7404, and 5 µM mindeudesivir, and infected with the EV11 or mock. After 72 h, fluorescent and bright-field microscopic images of eGFP-expressing organoids were taken. Scale bar 1 mm. (**b**) CTG reagent was added to the organoids and luminescence was measured. The responses were normalized to mock-infected vehicle-treated control. Mean ± SD, *n* = 3 (**c**) Median tissue culture infectious dose (TCID_50_) was determined from organoids media obtained 72 h post infection before addition of CTG reagent (mean, technical replicate *n* = 2, maximum assay limit 3e^5^ TCID_50_/mL). (**d**) Organoids were treated with increasing concentrations of pleconaril, AG7404, mindeudesivir or their combinations, and infected with the EV11 (moi 0.1). After 72 h, viability of virus-infected cells was determined using a CTG assay. The 4 × 4 × 4 interaction landscapes were obtained for the combination, and Bliss synergy scores were calculated

**Fig. 6:**
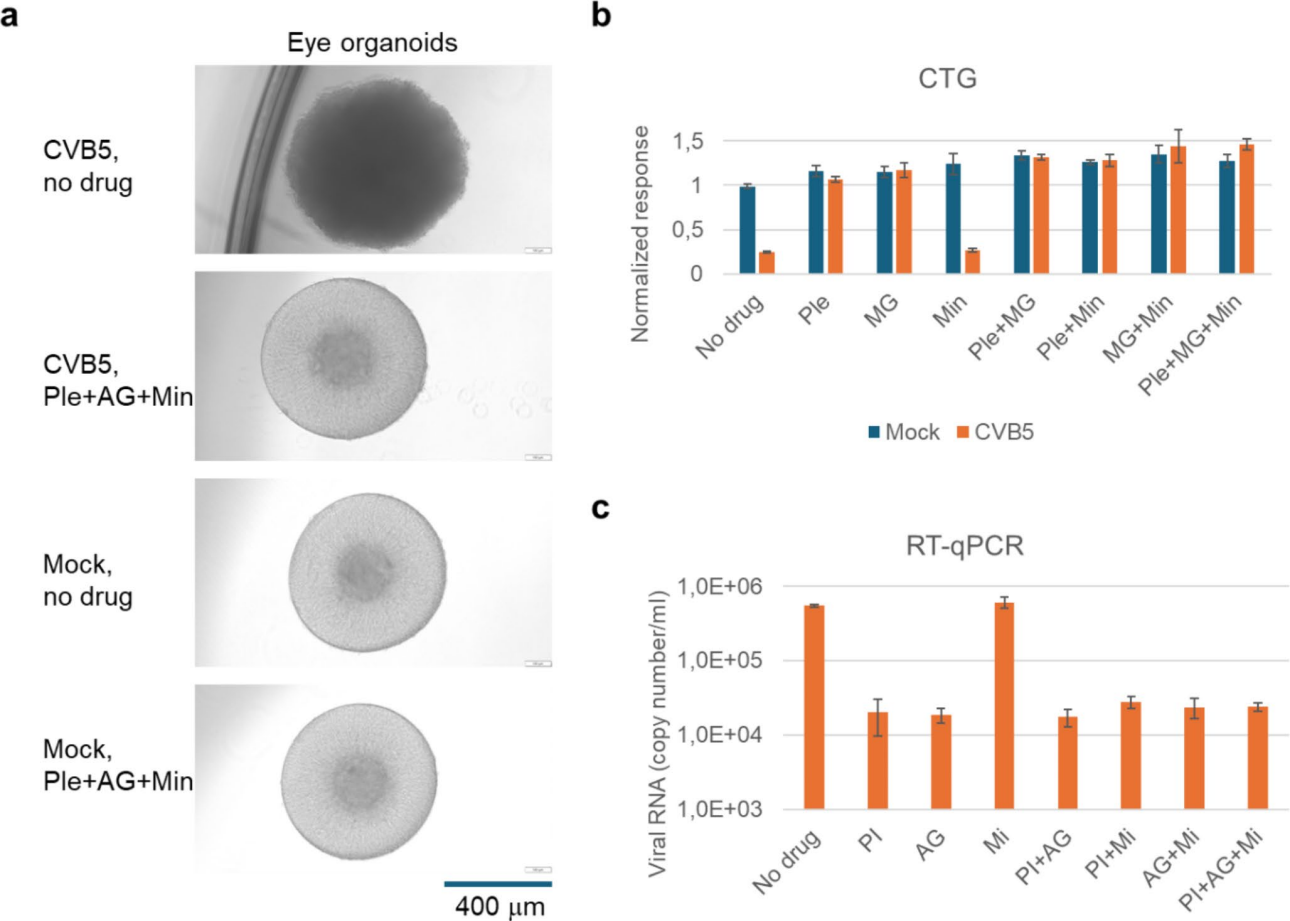
Effect of pleconaril, AG7404, and mindeudesivir combination on CVB5- and mock-infected brain organoids. (**a**) Organoids were differentiated for 30 days, treated with combination of 0.1 µM pleconaril, 1 µM AG7404, and 10 µM mindeudesivir, and infected with the CVB5 (moi 0.1) or mock. After 1 h of infection, the media was replaced with fresh media containing the drugs but not the virus. After 72 h, CellToxGreen (CTxG, 1:1000) was added to visualize dead cells, and microscopic images of the organoids were taken. Scale bar, 1 mm. (**b**) Organoids were treated with 0.1 µM pleconaril, 1 µM AG7404, and 10 µM mindeudesivir or their combinations for 15 min, and infected with the CVB5 or mock. After 72 h, CTG reagent was added, luminescence was measured, and the responses were normalized to mock-infected vehicle-treated controls. Mean ± SD, *n* = 3. (**c**) Organoids were treated as in (a). After 72 h, RT-qPCR analysis of viral RNA isolated from media and organoids was performed. Mean ± SD, *n* = 3

**Fig. 7:**
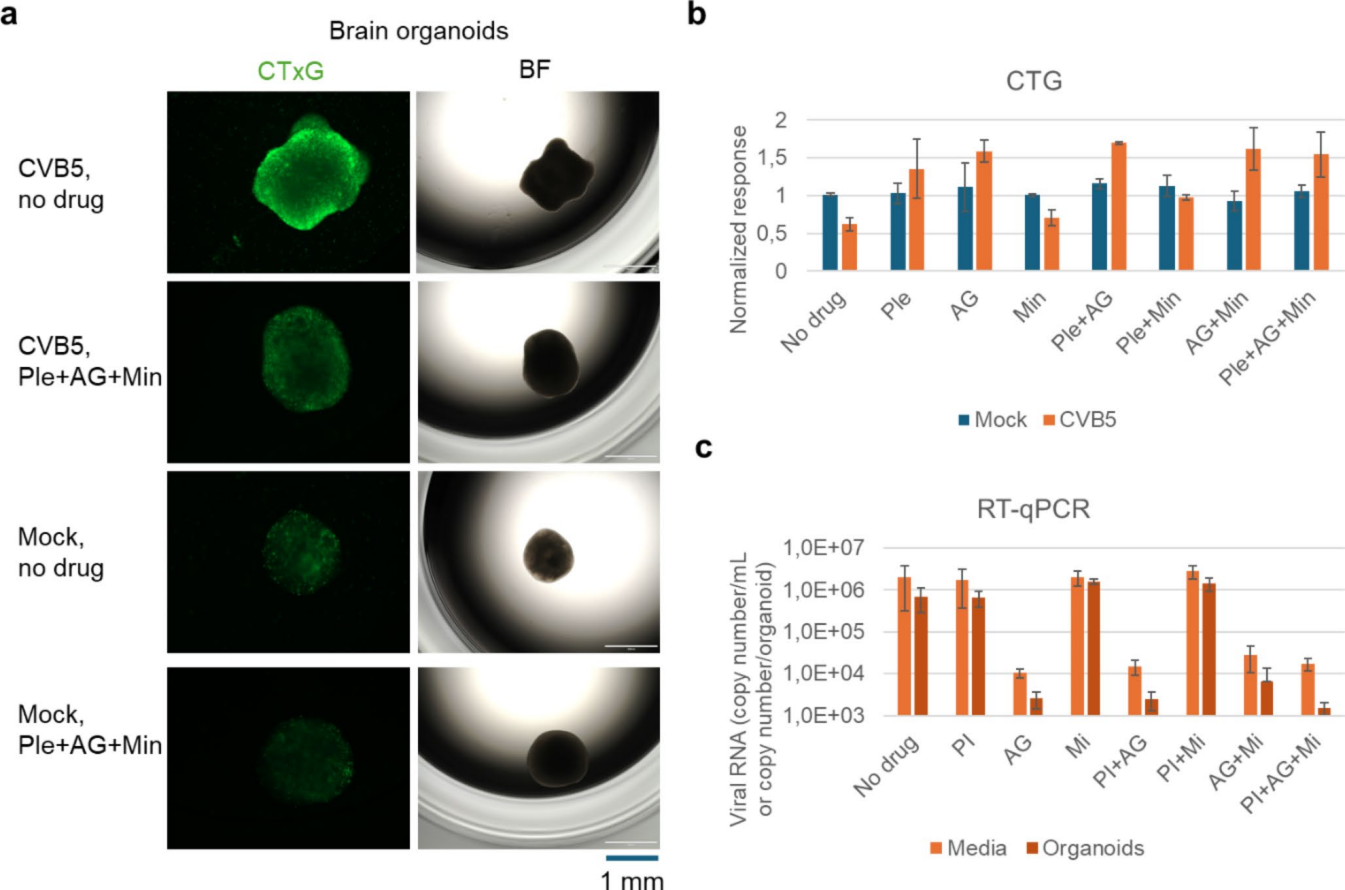
Effect of combination of pleconaril, AG7404, and mindeudesivir on CVB5- and mock-infected heart organoids. (**a**) Organoids were differentiated for 53 days, treated with combination of 0.1 µM pleconaril, 1 µM AG7404, and 10 µM mindeudesivir, and infected with the CVB5 or mock. 1 h after infection, the media was replaced with fresh media containing the drugs but not the virus. After 72 h, microscopic images of organoids were taken. Scale bar, 400 mm. (**b**) Differentiated organoids were treated with 0.1 µM pleconaril, 1 µM AG7404, and 10 µM mindeudesivir or their combinations for 15 min, and infected with the CVB5 or mock. After 72 h, movies of virus- and mock-infected organoids were recorded and transformed into cardiograms. (**c**) Organoids were treated and infected as in (a). After 72 h, RT-qPCR analysis of viral RNA isolated from media of CVB5-infeced organoids was performed. Mean ± SD, *n* = 3

**Fig. 8:**
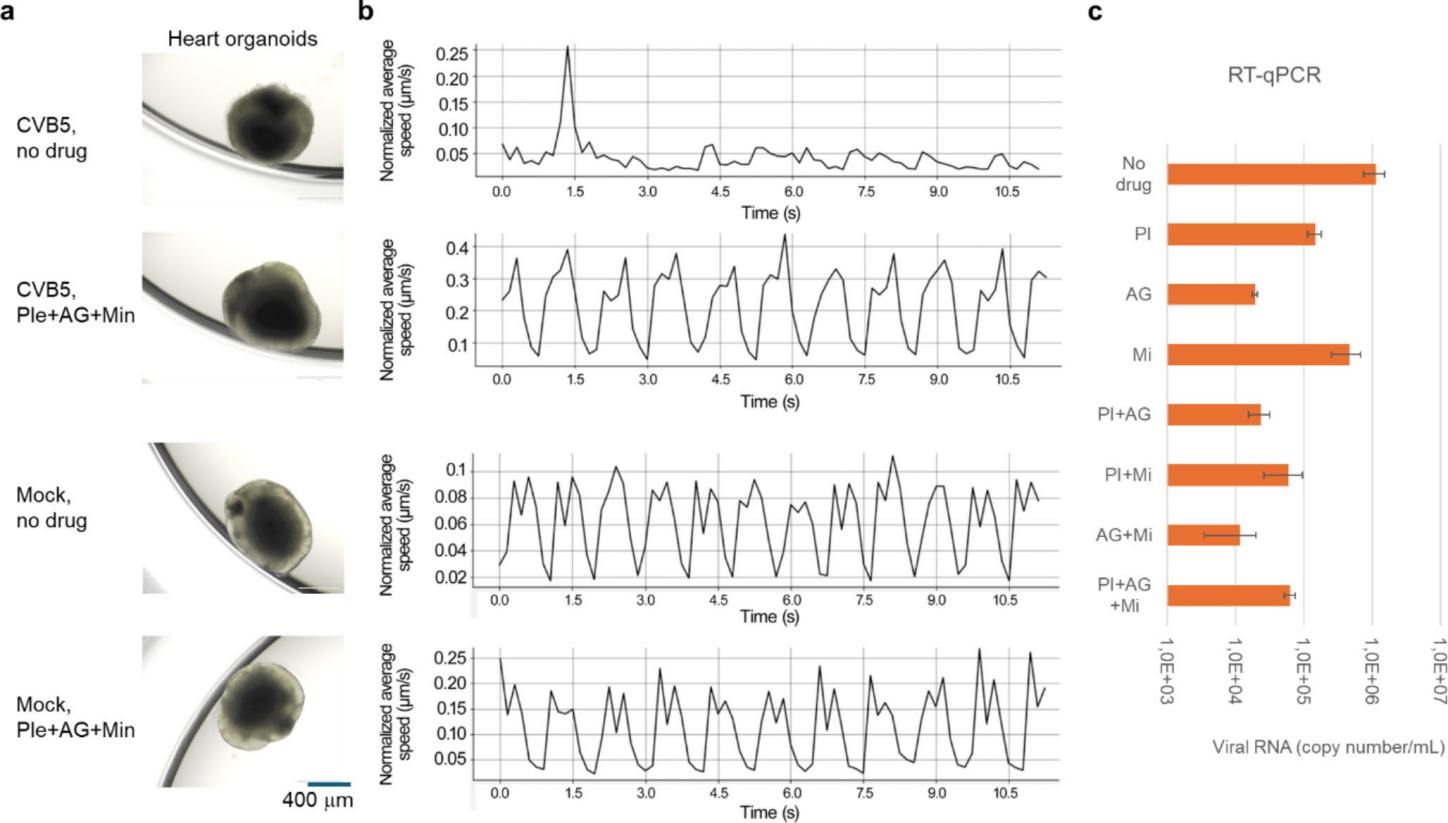
Effect of pleconaril, AG7404, and mindeudesivir combination on CVB5- and mock-infected eye organoids. (**a**) Organoids were differentiated for 20 days, treated with combination of 0.1 µM pleconaril, 1 µM AG7404, and 10 µM mindeudesivir, and infected with the CVB5 or mock. 1 h after infection, the media was replaced with fresh media containing the drugs but not the virus. After 72 h, microscopic images of organoids were taken. Scale bar, 400 mm. (**b**) Organoids were treated with 0.1 µM pleconaril, 1 µM AG7404, and 10 µM mindeudesivir or their combinations for 15 min, and infected with the CVB5 or mock. After 72 h, CTG reagent was added, luminescence was measured and the responses were normalized to mock-infected, non-treated controls. Mean ± SD, *n* = 3. (**c**) Organoids were treated and infected as in (a). After 72 h, RT-qPCR analysis of viral RNA isolated from media of CVB5-infeced organoids was performed. Mean ± SD, *n* = 3

We evaluated the effectiveness of the combination of pleconaril, AG7404, and mindeudesivir against enterovirus infections in human organoid systems, which provide a more accurate simulation of viral diseases compared to traditional cell cultures [35]. Given that enteroviruses can infect multiple organs, we generated various organoid systems representing some of these organs. Importantly, the combination at selected concentrations of drugs was not toxic for the organoids (Fig. S14-16, Figs. 5, 6, 7 and 8).

First, we generated organoids from the pancreatic cancer cells of two patients, designated PO34T and PO80T [25]. We found that EV1, EV6, EV11, and CVB5 successfully replicated in the eGFP-expressing pancreatic cancer organoids (Fig. S14, S15). EV6 and EV11 induced greater visible and detectable cytopathic effects than EV1 and CVB5, with PO80T being more sensitive to the viruses than PO34T. In both PO34T and PO80T, the combination of pleconaril, AG7404, and mindeudesivir effectively rescued the organoids from EV6/EV11-mediated death and significantly reduced viral replication (Fig. 5a-c). We also conducted synergy test using multiple doses of drugs Fig. 5d shows that the combination demonstrated synergistic/additive effect.

Second, we generated human airway organoids (hAOs) from tumor-free material from the lung of a cancer patient. We tested the efficacy of the combination of pleconaril, AG7404, and mindeudesivir in the organoids against EV1. The drugs, in combination at non-cytotoxic concentrations, significantly reduced viral replication, as indicated by RT-qPCR analysis of viral RNA extracted from the media and organoids (Fig. S16).

Finally, we generated eye, brain and heart organoids from iPSCs derived from healthy donor. We found that CVB5 replicated more efficiently in eye and brain organoids than EV1, EV6, or EV11 whereas all four viruses replicated in heart organoids (Fig. S17). We evaluated the efficacy of the combination of pleconaril, AG7404, and mindeudesivir against CVB5 in the organoids. The combination preserved the integrity of all infected organoids and attenuated viral replication (Figs. 6, 7 and 8). Importantly it rescued the brain and eye organoids from virus-mediated cell death. Moreover, the cocktail preserved contraction rhythm of infected heart organoids (Movies S1-4).

Thus, we identified a combination of orally available, safe-in-human pleconaril, AG7404, and mindeudesivir that inhibits a broad range of enterovirus infections in different human organoid systems.

## Discussion

Viral diseases remain a global economic and public health challenge, with many lacking effective treatments. Developing antivirals against viral diseases, which may lead to severe outbreaks, is particularly challenging. To date, antivirals have been approved primarily for the treatment of SARS-CoV-2, HCV, IAV, and HIV. Most of these antivirals are used in combination to combat the rapid emergence of viral drug resistance.

Here, we identified a drug cocktail containing safe-in-human, orally available pleconaril, AG7404, and mindeudesivir against enterovirus infections which inhibited enterovirus replication at non-toxic concentrations in human lung, eye, cervical, skeletal, and pancreatic cells. The combination protected human pancreatic, eye, lung, heart, and brain organoids from enterovirus-mediated death. Interestingly, it did not affect glucose metabolism and insulin secretion by pancreatic β-cells and preserved the contraction rhythm of virus-infected heart organoids. Thus, this combination consistently exhibits additive or synergistic effects across a range of enteroviruses in vitro, emphasizing its potential as a broad-spectrum antiviral cocktail. Additionally, the multi-stage action on viral replication suggests that the combination could remain effective even when administered post-infection, with no detectable loss of activity.

In our previous work with the pleconaril-rupintrivir-remdesivir combination [13], we observed that resistance to the triple-drug regimen developed more slowly compared to double combinations or single agents. This was due to the virus needing to accumulate multiple mutations in key viral factors, such as VP1, 3CPro, and Pol, to overcome the drug cocktail. We hypothesize that our new combination, consisting of pleconaril, AG7404, and mindeudesivir, each targeting these critical viral factors, may offer a similar advantage. Further development of this combination, both in vitro and in vivo against various enteroviruses, as well as in clinical trials, could provide a potential treatment solution for diseases associated with enteroviruses, including T1D, hepatitis A, and HFMD [33, 37, 38].

Furthermore, this combination could be used to treat viral co-infections. Mindeudesivir has been shown to inhibit RSV and coronaviruses, while AG7404 has been shown to inhibit SARS-CoV-1 and SARS-CoV-2 [36, 39, 40]. Therefore, the combination could be exploited for treatment of the virus co-infections.

Our study emphasizes the potential of orally available, synergistic broad-spectrum drug cocktails with high resistance barriers as strategic tools for managing emerging and reemerging viral diseases. These include but are not limited to viral hemorrhagic fevers (RVFV, CHHF, LASV, DenV, YFV, EboV, MarV, etc.), oral and genital herpes (HSV-1 and − 2), chickenpox and shingles (VZV), and certain cancers (KSHV, EBV). Furthermore, commercially available combinations for treating AIDS (HIV), COVID-19 (SARS-CoV-2), and viral hepatitis (HCV, HBV) could be optimized to reduce side effects and increase efficacy. To optimize existing and develop novel combinations, several key concepts should be leveraged: (i) use antivirals that target a broad range of viruses; (ii) Use antiviral cocktails that slow down the development of viral drug resistance; (iii) utilize drug combination synergy principles to enhance efficacy and reduce toxicity; (iv) Use antivirals in combinations that can be delivered via the same route; (v) employ different organoid systems corresponding to virus tropism to demonstrate antiviral efficacy and assess drug impacts on organ function. This approach could serve as a general paradigm for the effective development of new combinational drug therapies. The translational potential of such an approach is underscored by its ability to save significant time and costs, given that orally available, safe-in-human counterparts of the cocktails are already at advanced developmental stages.

## Electronic supplementary material

Below is the link to the electronic supplementary material.

Supplementary Material 1

Supplementary Material 2

Supplementary Material 3

Supplementary Material 4

Supplementary Material 5

## Data Availability

All data generated or analyzed during this study are included in this published article and its supplementary information files.
