# Supplementary material for: Synergistic combination of orally available safe-in-man pleconaril, AG7404, and mindeudesivir inhibits enterovirus infections in human cell and organoid cultures
Source: Cell Mol Life Sci. 2025 Jan 23;82(1):57. doi: 10.1007/s00018-025-05581-4 (PMC11754576; doi:10.1007/s00018-025-05581-4)
Supplement: Supplementary file 5 — Supplementary Material 5 [file 18_2025_5581_MOESM5_ESM.docx]

**Supplementary tables and figures**

Table S1. Chemical compounds used as antivirals in this study.

| **Compound** | **Oral** | **Supplier** | **Catalog N.** | **Cas N.** |
| --- | --- | --- | --- | --- |
| Pleconaril | Yes | Cayman Chemical | CAYM28461 | 153168-05-9 |
| Vapendavir | Yes | MedChemExpress | HY-106254A | 439085-51-5 |
| Rupintrivir | No | Sigma-Aldrich | PZ0315-5MG | 223537-30-2 |
| AG7404 | Yes | MEDKOO | 530656 | 343565-99-1 |
| Remdesivir | No | Cayman Chemical | 30354 | 1809249-37-3 |
| Mindeudesivir | Yes | MedChemExpress | HY-145119AS | 2779498-79-0 |
| Obeldesivir | Yes | MedChemExpress | HY-145994 | 2647441-36-7 |
| LY2334737 | Yes | MedChemExpress | HY-13672 | 892128-60-8 |
| Favipiravir | Yes | MedChemExpress | HY-14768 | 259793-96-9 |
| Ribavirin | Yes | MedChemExpress | HY-B0434 | 36791-04-5 |
| Sofosbuvir | Yes | MedChemExpress | HY-15005 | 1190307-88-0 |
| Molnupiravir | Yes | MedChemExpress | HY-135853 | 2492423-29-5 |

Table S2. Virus strains used in this study.

| **Virus name*** | **ICTV name**** | **ICTV species *** | **Reference** |
| --- | --- | --- | --- |
| EVA71 | EV-A71 | *Enterovirus alphacoxsackie* | ATCC VR-1775 |
| EV1 | E1 | *Enterovirus betacoxsackie* | ATCC VR-1808 |
| EV6 | E6 | *Enterovirus betacoxsackie* | (1) |
| EV7 | E7 | *Enterovirus betacoxsackie* | (2) |
| EV11 | E11 | *Enterovirus betacoxsackie* | (3) |
| CVA13 | CVA-13 | *Enterovirus coxsackiepol* | EVAg 014V-03623 |
| CVB5 | CVB5 | *Enterovirus betacoxsackie* | (3) |
| CVB6 | CVB6 | *Enterovirus betacoxsackie* | ATCC VR-155 |

* - Virus names are not official ICTV designations.

** - ICTV names are from <https://ictv.global/report/chapter/picornaviridae/picornaviridae/enterovirus>

Table S3. Cells used in this study.

| **Cells** | **Reference** |
| --- | --- |
| Human adenocarcinoma alveolar basal epithelial A549 cells | ATCC CCL-185 |
| Human pancreatic cancer MIA PaCa-2 cells | ATCC CRM-CRL-1420 |
| Human Lung Embryonal Fibroblast HE Cells | ATCC CCL-171 |
| Human rhabdomyosarcoma RD cells | ATCC CCL-136 |
| Human cervical carcinoma HeLa cells | ATCC CCL-2 |
| African green monkey kidney Vero cells | ATCC CCL-81 |
| Human immortalized retinal pigment epithelium RPE cells | CRL-4000 |
| Human beta Endoc-BH5 cells | Human Cell Design |
| Human iPS generated from ATCC dermal fibroblast | ATCC ACS-1011 |

**
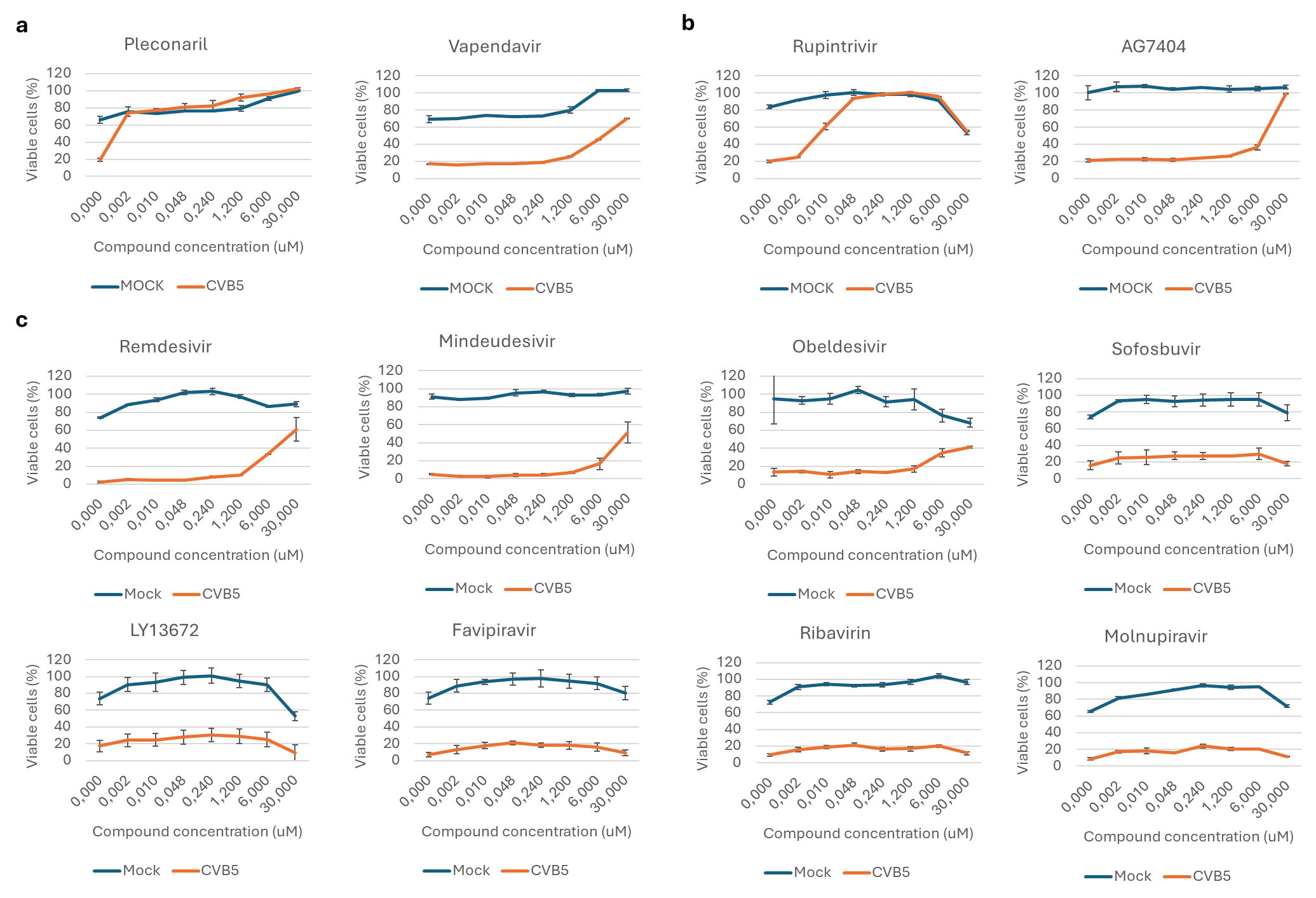
**

**Figure S1.** Efficacy and cytotoxicity of 12 compounds used in the study. (**a-c**) A549 cells were treated with increasing concentrations of (a) VP1 binders, (b) 3C Pro inhibitors, or (c) RNA synthesis inhibitors and then infected with CVB5 or mock-infected. After 48 hours, the viability of virus-infected and mock-infected cells was determined using a CTG assay. Mean ± SD, n=3.


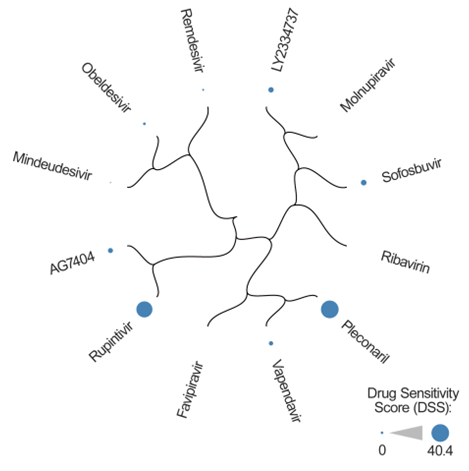


**Figure S2.** Structure-activity relationship (SAR) of 12 compounds used in the study. (**a**) Drug sensitivity scores (DSS) for both mock and virus conditions were calculated. The differences (ΔDSS) were displayed in the SAR diagram as bubbles. The size of the bubbles corresponds to the ΔDSS values of the compounds. Similarity between compounds in SAR diagram was calculated using ECPF4 fingerprints and visualized with the D3 JavaScript library.


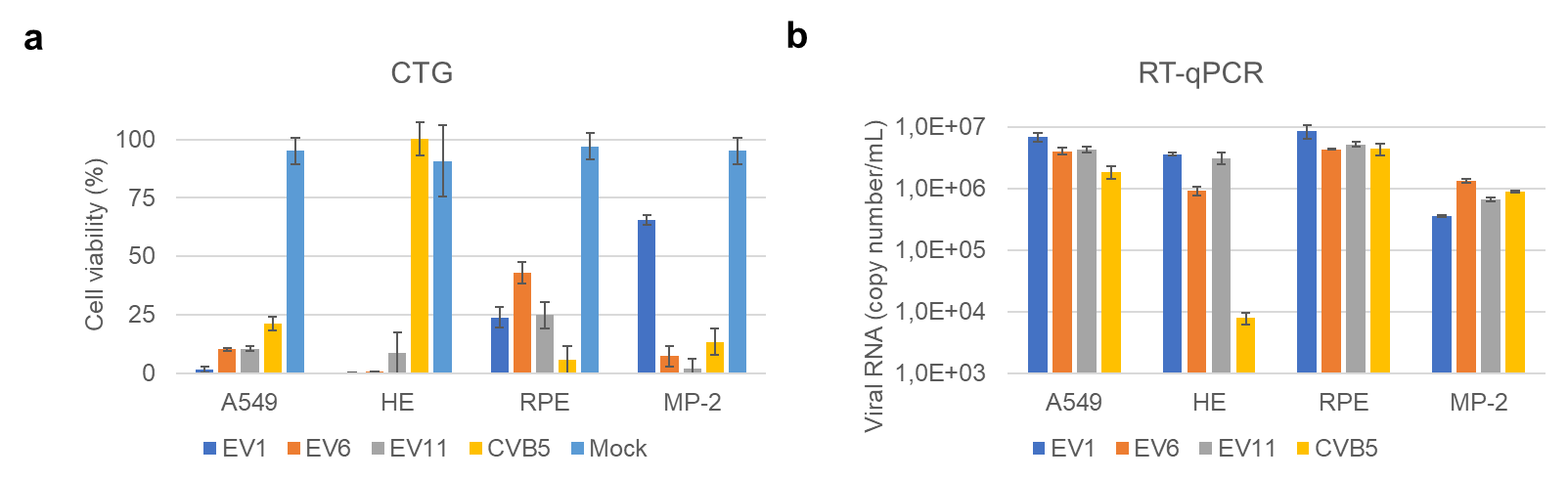


**Figure S3**. Susceptibility of 4 cell line for replication of 4 enteroviruses. (a) A549, HE, MiaPaca-2 (MP-2) and RPE cells were infected with of CVB5, EV1, EV6, EV11 (moi 0.1) or mock. After 48 hours, the cell viability was determined using a CTG assay. Mean ± SD, n=3. (b) RT-qPCR analysis of viral RNA isolated from media of infected cells. Mean ± SD; n=3.

**Figure S4.** Totoxicity and anti-enterovirus efficacy of VP1 binders (pleconaril and vapendavir), 3C protease inhibitors (rupintrivir and AG7404) and RNA synthesis inhibitors (remdesivir and mindeudesivir). A549 cells were treated with increasing concentration of compounds and infected with of CVB5, EV1, EV6 or EV11 (moi 0.1) or mock. After 48 hours, the cell viability was determined using a CTG assay, values were normalized and expressed as %. Mean ± SD, n=3.

**Figure S5.** Totoxicity and anti-enterovirus efficacy of VP1 binders (pleconaril and vapendavir), 3C protease inhibitors (rupintrivir and AG7404) and RNA synthesis inhibitors (remdesivir and mindeudesivir). HE cells were treated with increasing concentration of compounds and infected with of CVB5, EV1, EV6 or EV11 (moi 0.1) or mock. After 48 hours, the cell viability was determined using a CTG assay, values were normalized and expressed as %. Mean ± SD, n=3.

**Figure S6.** Totoxicity and anti-enterovirus efficacy of VP1 binders (pleconaril and vapendavir), 3C protease inhibitors (rupintrivir and AG7404) and RNA synthesis inhibitors (remdesivir and mindeudesivir). RPE cells were treated with increasing concentration of compounds and infected with of CVB5, EV1, EV6 or EV11 (moi 0.1) or mock. After 48 hours, the cell viability was determined using a CTG assay, values were normalized and expressed as %. Mean ± SD, n=3.

**Figure S7.** Totoxicity and anti-enterovirus efficacy of VP1 binders (pleconaril and vapendavir), 3C protease inhibitors (rupintrivir and AG7404) and RNA synthesis inhibitors (remdesivir and mindeudesivir). MP-2 cells were treated with increasing concentration of compounds and infected with of CVB5, EV1, EV6 or EV11 (moi 0.1) or mock. After 48 hours, the cell viability was determined using a CTG assay, values were normalized and expressed as %. Mean ± SD, n=3.

**
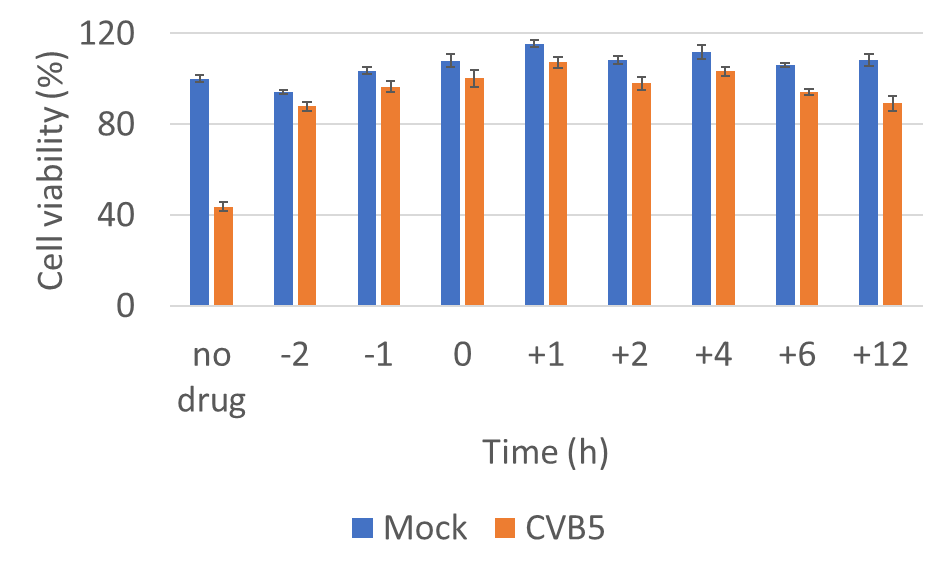
**

**Figure S8.** The time-of-addition experiment. A549 cells were treated with 0.1 μM pleconaril, 1 μM AG7404, and 10 μM mindeudesivir at -2, -1, 0 (infection), +1, +2, +4, +6, +12 h before or after infection with CVB5 (moi 0.1) or mock. After 48 hours, the cell viability was determined using a CTG assay. Mean ± SD, n=3

**Figure S9**. A549 cells were treated with increasing concentrations of pleconaril, AG7404, mindeudesivir or their combinations, and infected with the mock or CVB5 (moi 0.1). After 48 h, cell viability was determined using a CTG assay values were normalized and expressed as %. The interaction landscapes are shown.

**Figure S10**. HE cells were treated with increasing concentrations of pleconaril, AG7404, mindeudesivir or their combinations, and infected with the mock or EV11 (moi 0.1). After 48 h, cell viability was determined using a CTG assay values were normalized and expressed as %. The interaction landscapes are shown.

**Figure S11**. RPE cells were treated with increasing concentrations of pleconaril, AG7404, mindeudesivir or their combinations, and infected with the mock or CVB5 (moi 0.1). After 48 h, cell viability was determined using a CTG assay values were normalized and expressed as %. The interaction landscapes are shown.

**Figure S12**. MP-2 cells were treated with increasing concentrations of pleconaril, AG7404, mindeudesivir or their combinations, and infected with the mock or CVB5 (moi 0.1). After 48 h, cell viability was determined using a CTG assay, values were normalized and expressed as %. The interaction landscapes are shown.


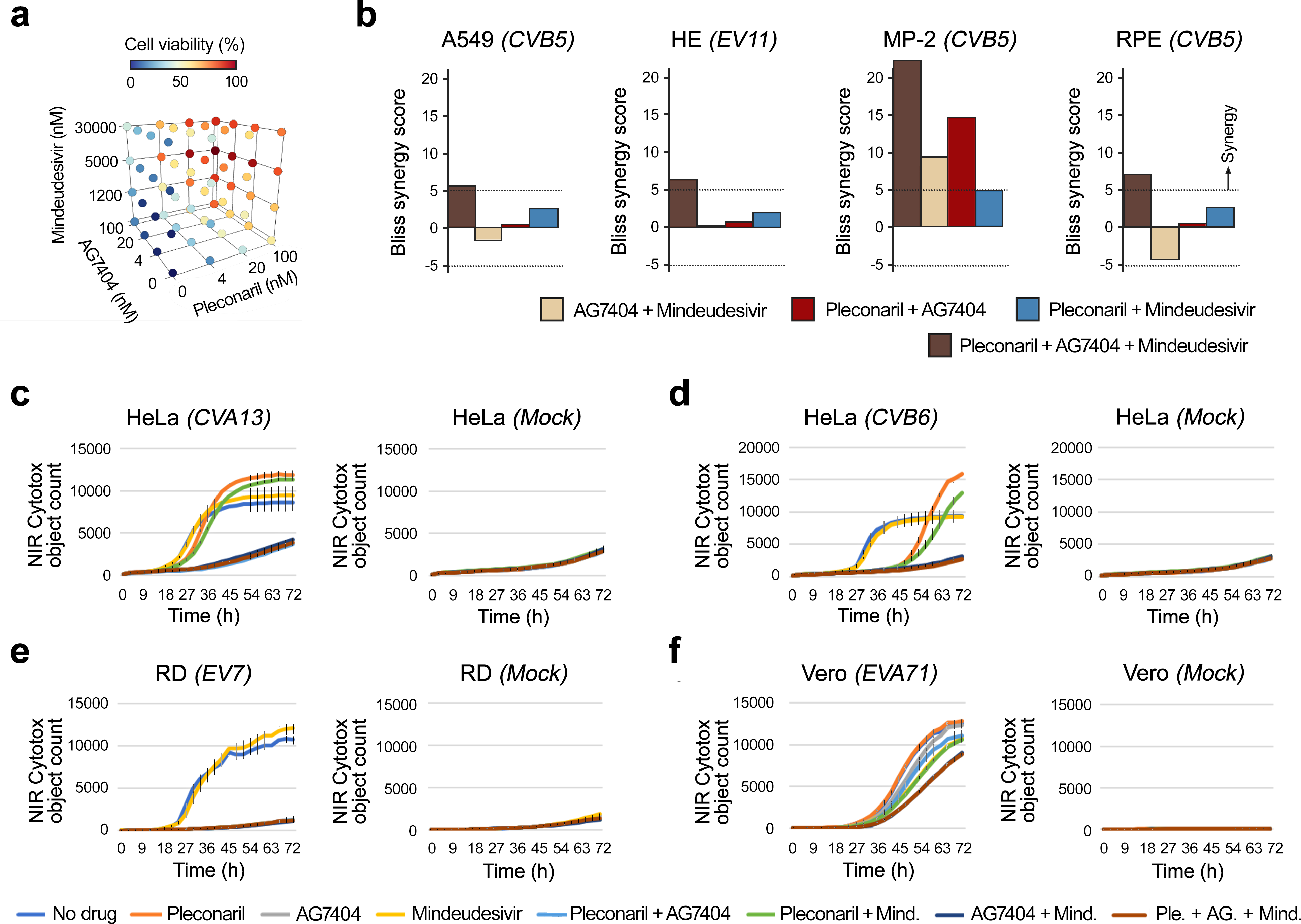


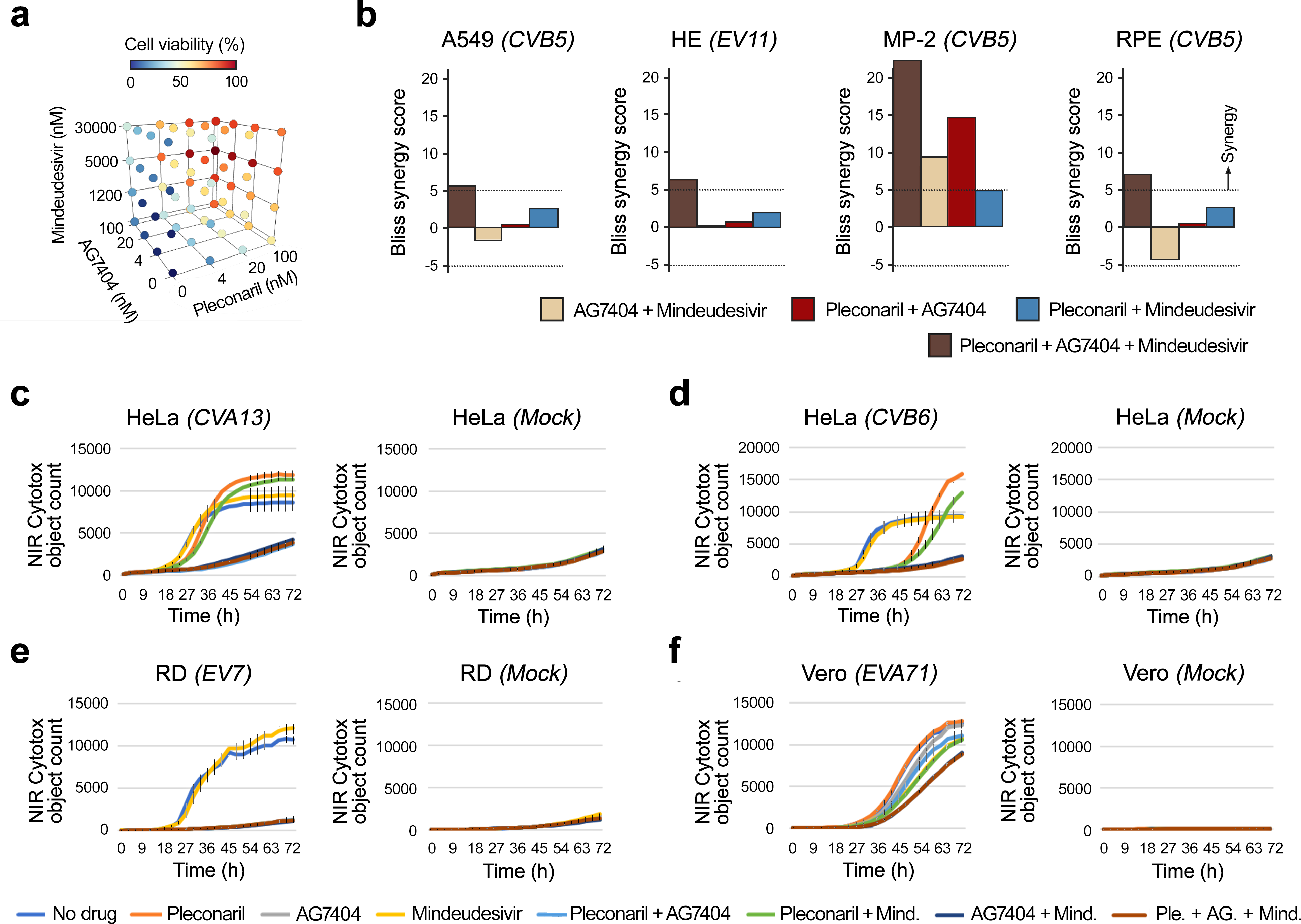


**Figure S13**. Anti-enteroviral effect of combination of pleconaril, AG7404 and mindeudesivir in cell cultures. Cells were treated with 0.1 μM pleconaril, 1 μM AG7404, 10 μM mindeudesivir or their combinations and infected with CVA13 (moi 0.1), CVB6 (moi 0.1), EV7 (moi 0.1), EVA71 (moi 0.1) or mock. NIR Cytotox dye was added upon infection, cells were imaged at 3-hour intervals for 72 h and NIR-positive (dead) cells were calculated. Mean±SD, n=3.

**
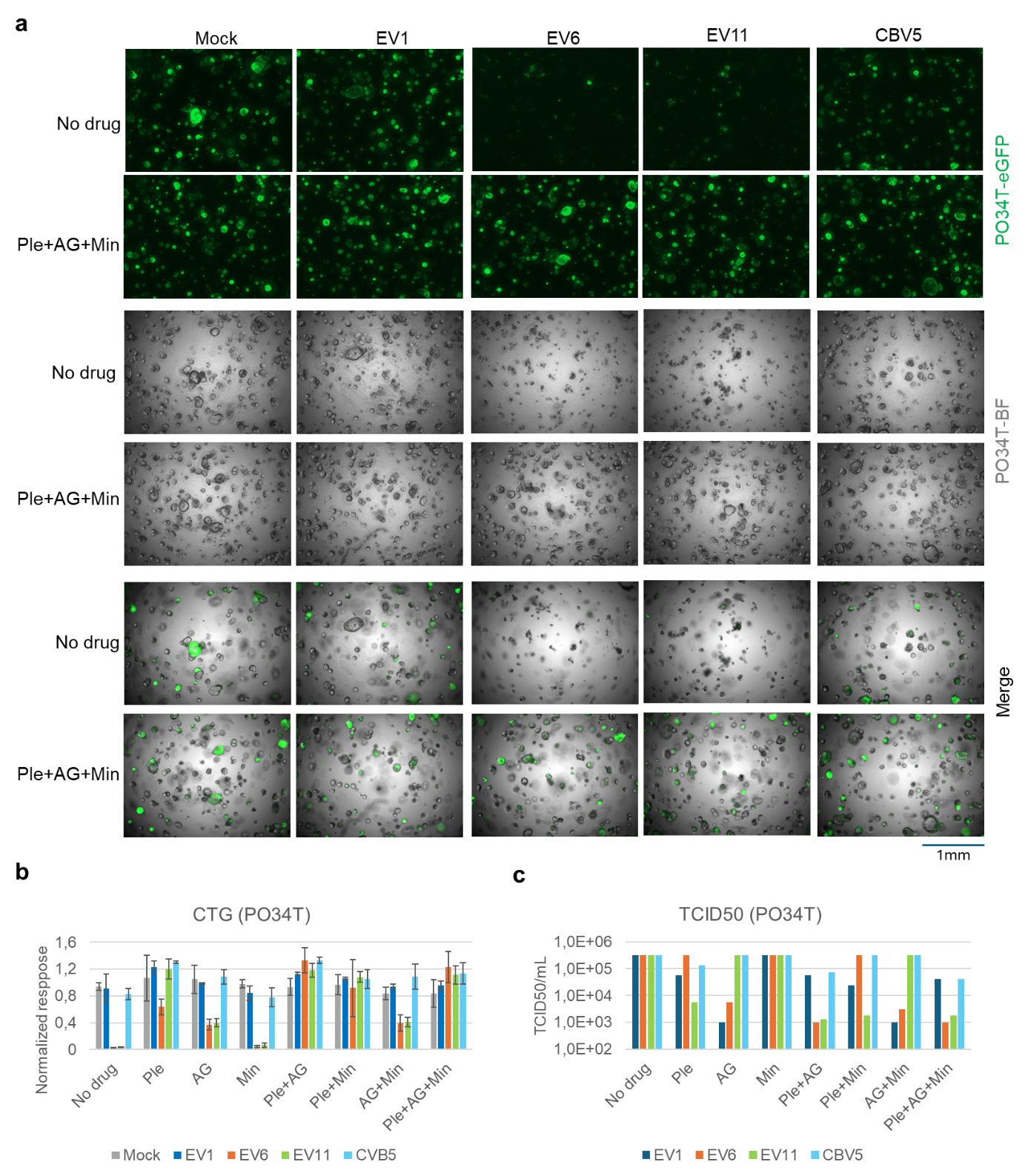
**

**Figure S14.** Effect of pleconaril, AG7404, and mindeudesivir combinations on virus- and mock-infected organoids derived from PO34T pancreatic cancer patient. (**a**) Organoids were differentiated for 4 days, treated with combination of 0.1 µM pleconaril, 5 µM AG7404, and 5 µM mindeudesivir, and infected with the viruses (1000 PFU/well) or mock. After 72 h, microscopic images of virus- and mock-infected organoids were taken. Scale bar, 1 mm. (**b**) After 72 h, CTG reagent was added, luminescence was measured and the responses were normalized to mock-infected, non-treated control. Mean ± SD, *n*=3. (**c**) After 72 h, before addition of CTG reagent, media were collected, and median tissue culture infectious dose (TCID_50_) was determined (maximum assay limit 300000 TCID50/ml).

**
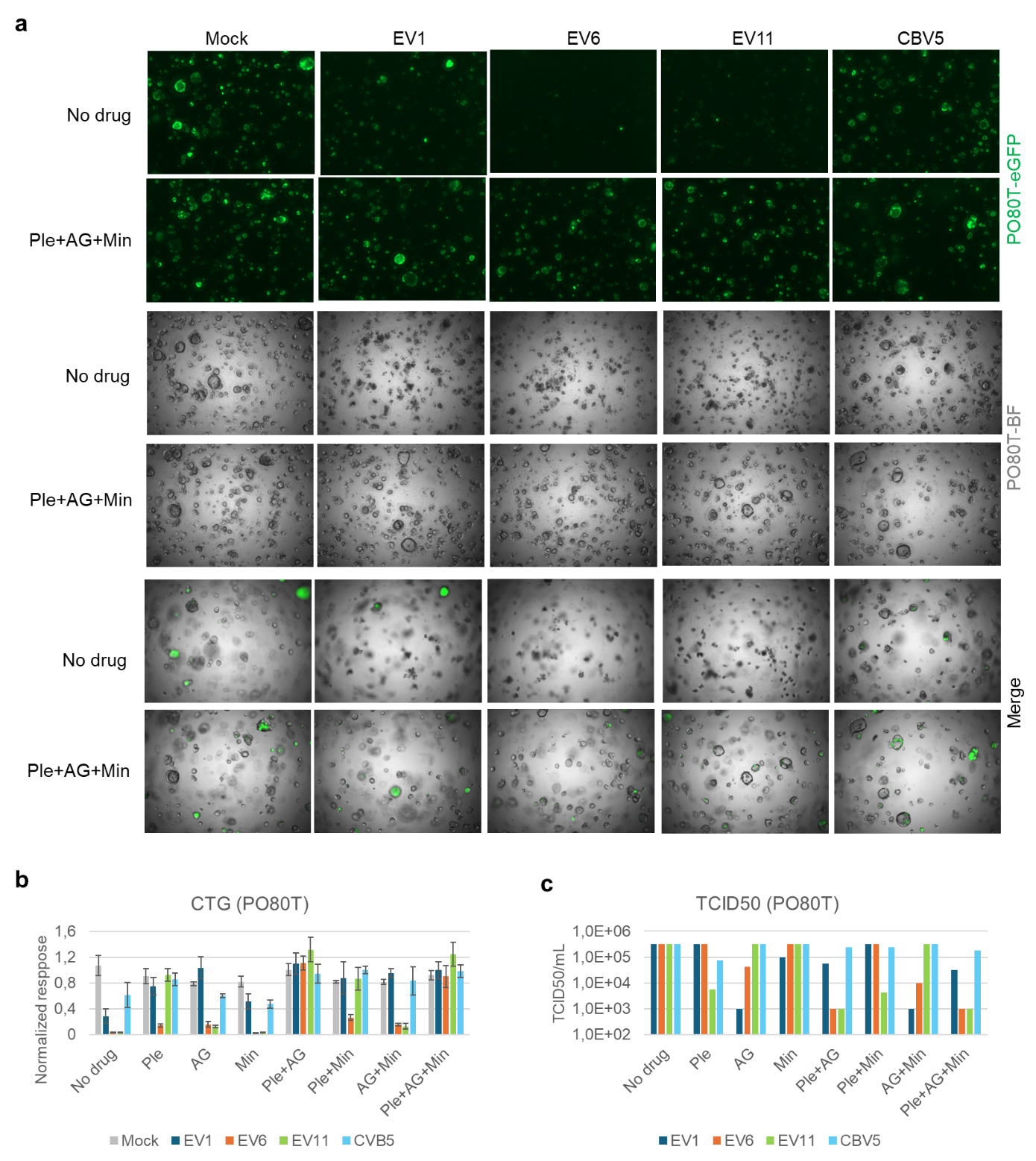
**

**Figure S15.** Effect of pleconaril, AG7404, and mindeudesivir combinations on virus- and mock-infected organoids derived from PO80T pancreatic cancer patient. (**a**) Organoids were differentiated for 4 days, treated with combination of 0.1 µM pleconaril, 5 µM AG7404, and 5 µM mindeudesivir, and infected with the viruses (1000 PFU/well) or mock. After 72 h, microscopic images of virus- and mock-infected organoids were taken. Scale bar, 1 mm. (**b**) After 72 h, CTG reagent was added, luminescence was measured and the responses were normalized to mock-infected, non-treated control. Mean ± SD, *n*=3. (**c**) After 72 h, before addition of CTG reagent, media were collected, and median tissue culture infectious dose (TCID_50_) was determined (maximum assay limit 300000 TCID50/ml).

**
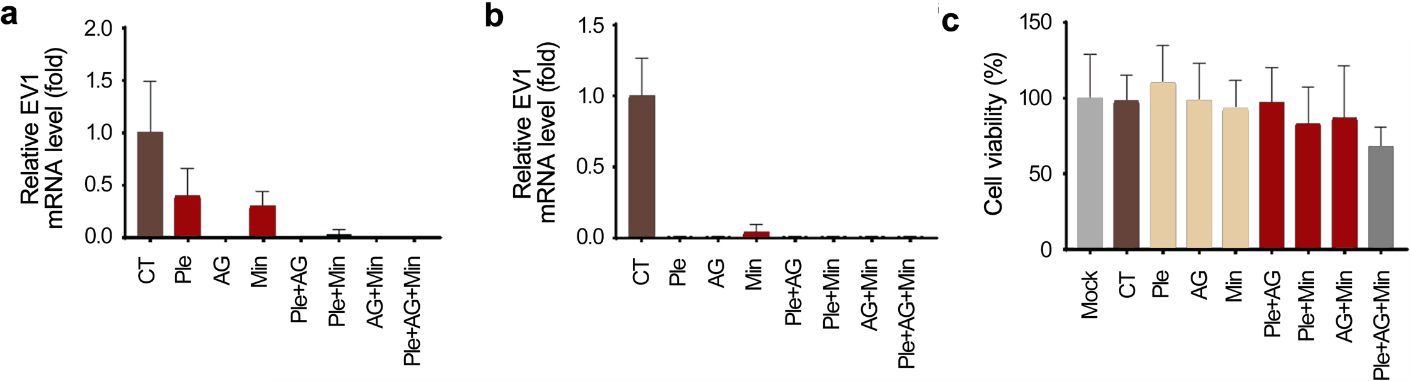
**

**Figure S16.** Effect of pleconaril, AG-7404 and mindeudesivir and their combinations on replication of EV1 in human airway organoids (hAOs). hAOs were infected with EV1 or mock and treated 0.1 μM pleconaril, 5 μM AG-7404, 5 μM mindeudesivir and their combinations. (a) RT-qPCR analysis of EV1 genes in HAOs was performed (n=4). (b) RT-qPCR analysis of EV1 genes in culture supernatant was performed (*n*=4). (c) Viability of virus- and mock-infected HAOs was determined using an Alamar Blue assay (*n*=4).


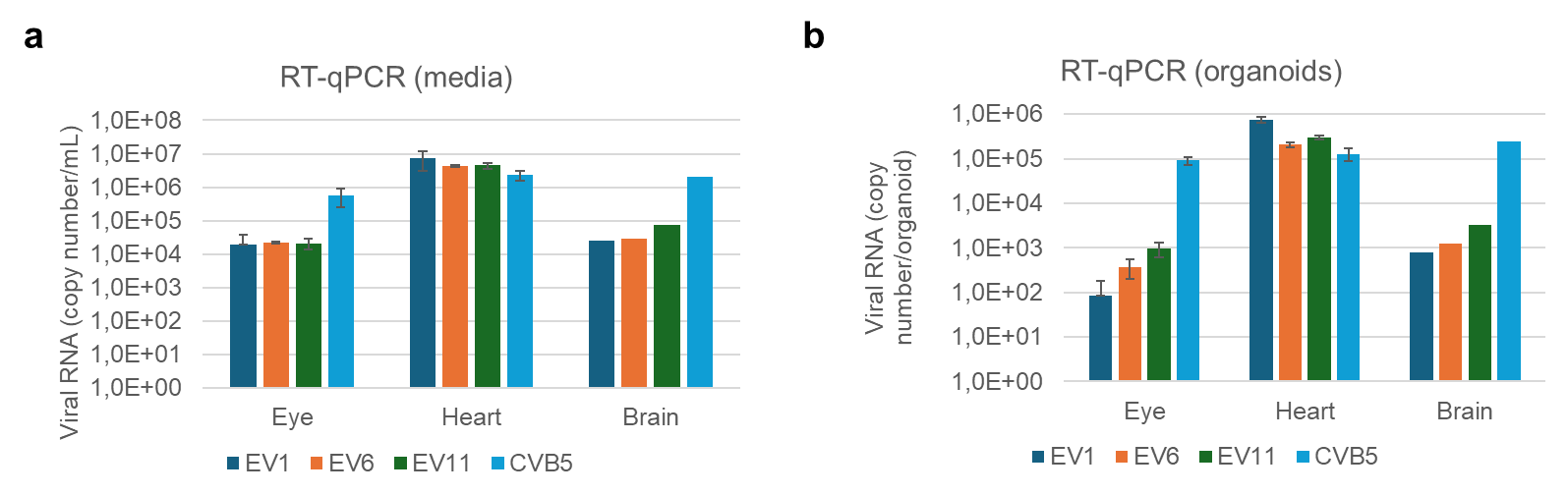


**Figure S17.** Replication of 4 enteroviruses in retinal (mean ± SD, *n=3*), heart (mean ± SD, *n=3*), and brain organoids assessed by RT-qPCR using the RNA extracted from (**a**) culture media and (**b**) organoids after 72 h of infection.

**References**

1. Smura, T., Kakkola, L., Blomqvist, S., Klemola, P., Parsons, A., Kallio-Kokko, H. *et al.* (2013) Molecular evolution and epidemiology of echovirus 6 in Finland Infect Genet Evol **16**, 234-247 10.1016/j.meegid.2013.02.011

2. Lulla, V., Dinan, A. M., Hosmillo, M., Chaudhry, Y., Sherry, L., Irigoyen, N. *et al.* (2019) An upstream protein-coding region in enteroviruses modulates virus infection in gut epithelial cells Nat Microbiol **4**, 280-292 10.1038/s41564-018-0297-1

3. Ianevski, A., Froysa, I. T., Lysvand, H., Calitz, C., Smura, T., Schjelderup Nilsen, H. J. *et al.* (2024) The combination of pleconaril, rupintrivir, and remdesivir efficiently inhibits enterovirus infections in vitro, delaying the development of drug-resistant virus variants Antiviral Res **224**, 105842 10.1016/j.antiviral.2024.105842
